# The effects of exercise and diet on olfactory capability in detection dogs*

**DOI:** 10.1017/jns.2014.35

**Published:** 2014-10-13

**Authors:** Craig T. Angle, Joseph J. Wakshlag, Robert L. Gillette, Todd Steury, Pamela Haney, Jay Barrett, Terrence Fisher

**Affiliations:** 1 Auburn University Animal Health and Performance Program, Auburn University, Auburn, AL 36849, USA; 2 Department of Clinical Sciences, Cornell University College of Veterinary Medicine, Ithaca, NY 14853, USA; 3 Auburn University School of Forestry and Wildlife Sciences, Auburn University, Auburn, AL 36849, USA

**Keywords:** Olfaction, Diet, Exercise, Detection dogs, Explosives, CO, maize oil, EDD, explosive detection dog, ME, metabolisable energy, SP, smokeless powder

## Abstract

A previous work suggests that dietary fat may influence canine olfaction. The present study evaluated whether olfactory performance could be influenced by forms of dietary fat and exercise. Seventeen certified detection dogs were fed three different diets (high fat, low fat or high polyunsaturated fat) for 12 weeks. After 12 weeks, olfactory testing was performed using a scent wheel in an olfaction laboratory using three explosive materials. The dogs completed eight to twelve scent trials before and after a 30 min treadmill exercise on five consecutive days. A mixed-effect logistic regression model was used to examine how diet, pre- or post-exercise, trial number, odourant, mass of target and target position influenced the probability of dogs alerting on the target odour. There were no significant changes in the dog's ability to find a target odour at threshold amounts. Dogs were 1·42 (1·08, 1·87; 95 % CI) times as likely to find a target on the high polyunsaturated fat diet relative to the high-fat diet (*P* = 0·009). The low-fat diet was not significantly different from either the high-fat diet or the high polyunsaturated fat diet (*P* = 0·12). Dogs were 1·49 (1·26, 1·76; 95 % CI) times as likely to find a target prior to exercise relative to after exercise (*P* < 0·001). Dogs on the high PUFA diet utilising maize oil showed mild improvement in olfaction. The exact reasons are unknown; however, the higher relative amount of linoleic acid in the diet may play a role in olfactory sensation which warrants further examination of optimal diets for detection dogs.

Research on canine performance has long focused on energy and metabolism during different types of athletic performance^(^^1^^,^^2^^)^. There has been little focus on the abilities and needs associated with hunting and detection dogs who not only require optimal energetics and conditioning to perform their typical activities, but also optimal scenting capabilities. Much of the previous research into feeding dogs of this capacity (low-level endurance) stem from ideas to promote stamina in sled dogs^(^^3^^)^. At present, there are two published studies showing differences on olfactory capabilities in hunting dogs when altering diets^(^^4^^,^^5^^)^. Davenport *et al.*^(^^4^^)^ suggested that bird find rates were superior when utilising a diet that may have had superior digestibility and slightly higher fat concentration, whereas a second study by Altom^(^^5^^)^ suggested that fat sources might influence olfaction with medium and polyunsaturated TAG proving medium-chain TAG to be inferior to maize and animal-based fats.

The use of higher fat often comes at a cost of other macronutrients, including protein or carbohydrate. A previous work in endurance sled dogs suggested that higher protein diets may be preferred since they have the capacity to maintain plasma volume and haematocrit^(^^6^^,^^7^^)^. In addition, when dietary protein is reduced below 24 % metabolisable energy (ME) exercising sled dogs exhibited more musculoskeletal injuries and diminished maximal oxygen consumption suggesting that diets might be about 24 % ME or higher^(^^7^^)^. However, in practice, some handlers will add additional fat and/or meat sources to kibble ration which can dilute down either carbohydrate or protein energies in the diet. These practices may be creating changes in macronutrients that would be considered detrimental to performance. Therefore our study set out to utilise commonly fed commercial dog foods with or without supplementation of oil to achieve the specific ME concentrations from protein, fat and carbohydrate. The purpose of the present study was to determine olfactory capability of dogs before and after exercise when fed diets that differed in macronutrient content and fatty acid composition.

## Experimental methods

### Subjects

A group of eighteen (Labrador Retrievers) healthy explosive detector dogs (EDD) were identified based on their abilities to conduct standard EDD searches (e.g. vehicle and building). The EDD also had to have the capability to find explosive amounts below 20 g of raw explosives, which is much less than the classified industry standards. There were six female and twelve male EDD in the present study. The mean weight (age) at the beginning of the study of the seventeen EED was 26·9 (4·9) kg and a mean of 24·8-month old, respectively, based on weekly weight and body condition score assessment. Mean kilojoule (kJ) consumption per day was 2005 (445) kJ. The mean weight at the end of the trial was similar at 27·4 (4·8) kg for all dogs. All dogs were maintained within a body condition score of 4–6 and all dogs maintained their respective body condition scores throughout the entire trial. The nature of the project, care and use of the EDD was approved by an Institutional Animal Care and Use Committee at Auburn University.

### Conditioning and training programme

All dogs were conditioned and trained throughout the study, including regular treadmill training. All dogs were trained and certified on smokeless powder (SP), trinitrolouene and ammonium nitrate whereby 100 % finds were observed on all dogs during field detection activities. All dogs were also trained to detect 20 g of target raw material or less in an odour sterile olfaction laboratory.

### Olfaction testing laboratory procedures

A 12 × 16 × 8 ft^3^ scent room was constructed. The room was outfitted with soundproofing insulation and all walls, ceiling and floor were sealed to prevent any currents from developing in the room. A vacuum system was placed in the four corners of the room to evacuate any contaminating odours allowing total room air evacuation if needed. Located in the centre of the room was a scent wheel that contained eight positions. Each of the eight positions was attached to a central sealed vacuum unit that allowed for air to be evacuated directly around each position. Each position was outfitted with clamps that would hold steel baskets. Inside the steel baskets were glass dishes that held the target, and visual and olfactory distractors (e.g. sugar, tea, nuts or mulch). A steel mesh was placed over the glass dish to prevent the dogs from coming in contact with the explosive or aid. The dogs detected amounts of explosives ranging from 20 g to 1 mg. SP was used in everyday training and as a motivator target, in the scent room, where the dogs were always rewarded for alerting on SP. Trinitrotoluene was only used in the scent room and dogs were not exposed to it in the field-training environment. Ammonium nitrate was used both in the scent room and in field training (Supplementary Fig. 1).

During an olfaction test a blinded handler brought a dog into one corner of the room and had the dog sit. The handler then presented the first position on the wheel and said ‘search’. The dog searched positions 1–8 in a counter-clockwise manner and off lead. The test moderator in the opposite corner of the room collected test data and directed the handler on whether or not to reward the dog. The dogs were on a variable reward system so not to manipulate their threshold (lowest amount detected by a particular dog) below testable limits. Dogs were required to complete eight to twelve scent trials (one attempt to find the target around the wheel) before and after 30 min of exercise on the treadmill. The dog was then taken out of the room and then recalled back into the room and the process was repeated again up to 12 times in approximately a 10 min or less time period.

Significant care was taken to inhibit or eliminate odour contamination: the air was vacuumed out of the room after each test. The test moderator and tray switcher wore nitrile exam gloves that were changed out in between each test to ensure contamination did not occur. Three new distractor baskets were brought in and randomly replaced on the scent wheel after each test. The target odour was also randomly replaced based on computer-generated randomisation between 1 and 8 after every test. The baskets were only used once for one test, and then they were washed using a commercial dish washer before being used again.

### Diet formulation

The diets were made to recapitulate common feeding practices using a moderate protein, low-fat, high-carbohydrate food Royal Canin 25 Medium Breed; a moderate protein, high-fat, low-carbohydrate food Royal Canin 4800; or a moderate protein, low-fat, high-carbohydrate food (Royal Canin 25 Medium Breed) with additional maize oil (CO) added. This last diet was designed to replicate the addition of a common, inexpensive, high polyunsaturated fat source to typical maintenance foods and to dilute the protein calories^(^^3^^)^. The average ME and other selected nutrient information for all three diets is represented in Table 1.

**Table 1.** Major energy substrates, PUFA, essential mineral and essential vitamin content per kilojoule (kJ)*

Amount (kJ)	HF	LF	CO
Protein (g)	282·9	274·6	178·9
Fat (g)	266·2	153·9	262·1
Total omega 6 (g)	59·1	26·6	52·8
Total omega 3 (g)	7·1	4·2	2·9
LA + AA (g)	58·2	27·0	52·4
EPA + DHA	2·5	3·7	2·5
Omega 6:3 ratio	8·4	6·4	18·2
Calcium (g)	13·9	12·9	8·5
Phosphorus (g)	10·0	8·6	5·6
Sodium (g)	369·8	3·8	2·5
Potassium (g)	7·4	7·6	5·0
Magnesium (mg)	696·0	857·0	565·6
Copper (mg)	27·7	21·3	14·1
Iron (mg)	209·8	158·0	104·3
Manganese (mg)	55·5	81·1	53·5
Zinc (mg)	198·8	242·6	160·1
Selenium (mg)	0·2	0·4	0·2
Iodine (mg)	3·2	5·9	3·9
Vitamin A (IU)	23 111·1	14 472·3	9551·7
Vitamin D_3_ (IU)	1109·3	814·3	537·4
Vitamin E (mg)	647·1	548·0	361·7
Thiamine (mg)	11·1	6·7	4·4
Riboflavin (mg)	6·5	7·5	4·9
Panthotenate (mg)	37·9	32·4	21·4
Pyridoxine (mg)	6·0	7·5	4·9
Niacin (mg)	25·0	53·7	35·4
Folic acid (mg)	1·1	1·6	1·1
Cyanocobalamin (mg)	0·27	0·07	0·05
Choline (mg)	2773·3	2521·7	1664·3

LF, low-fat, high-carbohydrate food; HF, high-fat, low-carbohydrate food; CO, maize oil; LA, linoleic acid; AA, arachidonic acid.

* Indicated the ratio represented is total omega 6:3 ratio as provided by the company with additions based on USDA database for CO.

### Crossover design

Dogs were blocked based on computer-generated randomisation into groups based on even sex representation between groups of six dogs in a 3 × 3 Latin Square design whereby dogs were provided one of three diets (Table 1) for a 12-week period of time to allow for metabolic adjustments to the diet (8 weeks). At the end of every 12 weeks, the testing consisted of treadmill running and scent room testing.

### Statistical analysis

A mixed-effects logistic regression model was used to examine the factors that influenced the probability of dogs alerting on the target; and factors considered included diet, pre- or post-exercise, trial number, odour, mass of target, target position (8). In the models, the random effect used was day nested in test phase nested in dog. A partial likelihood-ratio test was used to evaluate the significance of individual variables. All analyses were conducted in R version 2.13.1 (R Development Core Team 2011) using the package version 3.1–102^(^^8^^)^. To assess dietary influence on threshold of detection, the median threshold value for each scent was determined and compared using a Kuskal–Wallis ANOVA.

## Results

*Dogs*: All but one dog completed the entire study. The one dog that did not complete the study was dropped due to an acute cranial cruciate rupture within the first month of the trial making him unable to continue in the protocol.

### Diet and exercise

Dogs were 1·42 (1·082, 1·87; 95 % CI) times as likely to find a target at or near their threshold on the CO diet relative to the high-fat, low-carbohydrate food diet (*P* = 0·01). The low-fat, high-carbohydrate food diet was not significantly different from either the high-fat diet or the CO diet (*P* > 0·123). Dogs were 1·49 (1·26, 1·76; 95 % CI) times as likely to find a target prior to exercise relative to after exercise (*P* < 0·001). When examining detection thresholds there was no difference between dietary groups for all three scents (Table 2).

**Table 2.** Medians and ranges for detection of three different explosives (SP, smokeless powder; AN, ammonium nitrile; TNT, trinitroluene) at thresholds by dogs (*n* 17; each group) during three different dietary trials using medium breed twenty-five Royal Canin (low-fat, high-carbohydrate food (LF)), medium breed Royal Canin and maize oil (CO) and Royal Canin 4800 (high-fat, low-carbohydrate food (HF))

	SP (g)	AN (g)	TNT (g)
CO	0·015 (0·01–0·1)	0·1 (0·2–2·5)	0·025 (0·01–0·6)
LF	0·02 (0·001–0·6)	0·1 (0·01–2·5)	0·05 (0·004–0·6)
HF	0·015 (0·001–0·1)	0·15 (0·01–0·15)	0·05 (0·002–0·3)

No significant differences were observed between dietary groups.

### Test evaluation

To evaluate the effectiveness of the testing procedures, the statistical model analysed the effect of trial, mass of target, aid (test scent) position and test number. There was no significant effect of trial number or mass of target on the probability of dogs alerting on the target. Dogs were 1·23 (1·18, 1·28; 95 % CI) times as likely to alert on a target for each increase in aid position (*P* < 0·001) on the wheel. Dogs were 1·84 (1·39, 2·44; 95 % CI) times as likely to alert on a target in test 3 relative to test 2 (*P* < 0·001), and 2·06 (1·54, 2·74; 95 % CI) times as likely to alert on a target in test 4 relative to test 2 (*P* < 0·001). Tests 3 and 4 were not significantly different from each other.

### Odour

There was a significant effect of odour on the likelihood of success (*P* < 0·001). Dogs were 4·6 (3·08, 6·90; 95 % CI) times as likely to alert on SP compared with ammonium nitrate (*P* < 0·001). Dogs were also 1·18 (0·99, 1·40; 95 % CI) times as likely to alert on trinitrotoluene compared with ammonium nitrate, although differences were not statistically significant (*P* = 0·059). Dogs were 3·91 (2·61, 5·87; 95 % CI) times as likely to alert on SP compared with trinitrotoluene (*P* < 0·001).

## Discussion

The dietary interventions examined were designed to further define the role of polyunsaturated fat and decreased ME from protein its influence on olfaction as a follow-up to both Davenport and Altom's findings that olfaction may be influenced by dietary fat sources^(^^4^^,^^5^^)^. There was an increased probability of a dog finding a target on the CO diet relative to the high-fat, low-carbohydrate food diet at threshold. Of course, the highest polyunsaturated fat content was in the CO diet as seen in Table 1, based on the USDA database and feed company calculations. While the next highest in linoleic acid to total fat ratio was the low-fat, high-carbohydrate food diet and the third was the high-fat, low-carbohydrate food diet. Studies in rodents have shown that diets higher in PUFA can enhance olfactory capabilities and that these fatty acids incorporate into the nasal epithelium^(^^9^^,^^10^^)^. The limited research on this topic relates to rodent olfactory epithelial function which suggests that long-chain omega three fatty acids, including EPA, DHA and arachidonic acid seem to inhibit the potassium channel function allowing more sensitive depolarisation of the olfactory neurons leading to potentially heightened activity^(^^9^^)^. This may also be occurring in the dogs but cannot be confirmed by our study. More research needs to be conducted to understand the specific reason for the difference and whether it is related directly to linoleic acid's effects or potentially more arachidonic acid from linoleic acid elongation and conversion in the olfactory epithelium. Despite these interesting findings, it must be noted that the threshold for all of the dogs for all three raw material targets were well below the certification amount (averages between 20 and 100 mg) of 20 g. From a practical point of view, the dogs detection thresholds being this low makes the effects of diet insignificant unless the scent capabilities need to be in the low milligram quantities.

From a musculoskeletal perspective there were no differences noted in overall condition or performance of the dogs during this testing procedures. This must be recognised since previous studies in sled dogs have shown that musculoskeletal performance and injury was higher in groups of dogs being fed an 18 % ME diet^(^^7^^)^. From these and other studies it has been suggested that dogs receive minimally 24 % ME protein during athletic training to prevent musculoskeletal compromise and to maintain maximal oxygen consumption^(^^6^^,^^7^^)^. The discrepancies between the present study and those previously published may be due to the differences in type of activity since sled dogs likely undergo a more rigorous training and competitive endeavours than the average detection dog thereby not needing the same amount of protein for musculoskeletal integrity. It is also possible that the previous study by Reynolds *et al.*^(^^7^^)^ although randomised, not being a cross-over design allowed for the confounding variable of group selection biasing the 18 % ME group data towards more musculoskeletal issues.

Our study also examined how exercise influenced find rates. There were significant effects for exercise on the dog's ability to locate targets with increased find rates before a rigorous exercise bout, again with no differences in threshold variance due to exercise. This agrees with what Gazit and Terkel^(^^11^^)^ found when analysing detection dogs locating targets after a 20 min run on a treadmill. Their findings show that dogs had a decrease in find rates after exercise which is similar to our results. Gazit and Terkel explained that the decrease was due to an increase in panting; however, we found no effect on trial number, and panting dissipates over the trial time which indicates that there was not a panting recovery issue immediately post-treadmill in our study. Furthermore, their study was markedly different in that dogs were performing open area searches on lead resulting in more scent dispersal and possible missed scent, rather than a specific trained task-oriented search (e.g. searching the scent wheel) as performed in our study, which may be the reason for our dog's ability to find such low amounts of target materials. Other olfaction studies such as Sargisson and McLean^(^^12^^)^ have used scent wheels to reduce the amount of variables and standardise the detection task.

Our study also evaluated whether or not the type of odour played a role in target location success. There was a significant effect of odour on the likelihood of success. This was expected because certain odours have different methodological constraints placed on them for the study. However, it appears whether or not the odour was used in training does not influence target location in the scent room and volatility may be the reason for differences in levels of detection based on mass of the aid (i.e. the amount of the explosive). Lastly, the study evaluated the use of a scent wheel and scent room to evaluate the probability of detecting small weight explosives in a controlled laboratory setting. There was no significant effect of trial number. However, there was a significant effect for aid position and phase of the testing as well as variations in dogs. It is possible that they developed a pattern and process of elimination when taking the test over time and searched more intensely as each trial progressed. Gazit and Terkel found that detection rates increased as dogs advanced from target 1 to target 3. They also evaluated sniffing frequency at each position and found no significant effects^(^^11^^,^^13^^)^.

Overall the scent wheel test appears to provide a valid measure of olfaction for testing accuracy and factors that might affect olfactory capabilities. The findings regarding diet although significant and possibly important in optimising olfaction may be insignificant in the field considering the low levels of detection threshold experienced throughout the study. As expected, immediately after exercise dogs were less capable in their olfactory capabilities. Our findings suggest a lack of uniformity in capability of dogs, while task learning and methods of olfaction utilised over time may play a significant role in success. These findings make it prudent to teach dogs to perform their activities in ‘real field’ situations allowing the dogs to utilise and learn optimal ways to search environments to ensure success.

## Supplementary material

For supplementary material accompanying this paper visit http://dx.doi.org/10.1017/S2048679014000354. click here to view supplementary material
