# Supplementary material for: The effects of exercise and diet on olfactory capability in detection dogs
Source: J Nutr Sci. 2014 Oct 13;3:e44. doi: 10.1017/jns.2014.35 (PMC4473149; doi:10.1017/jns.2014.35)
Supplement: Supplementary file 1 [file S2048679014000354sup001.docx]

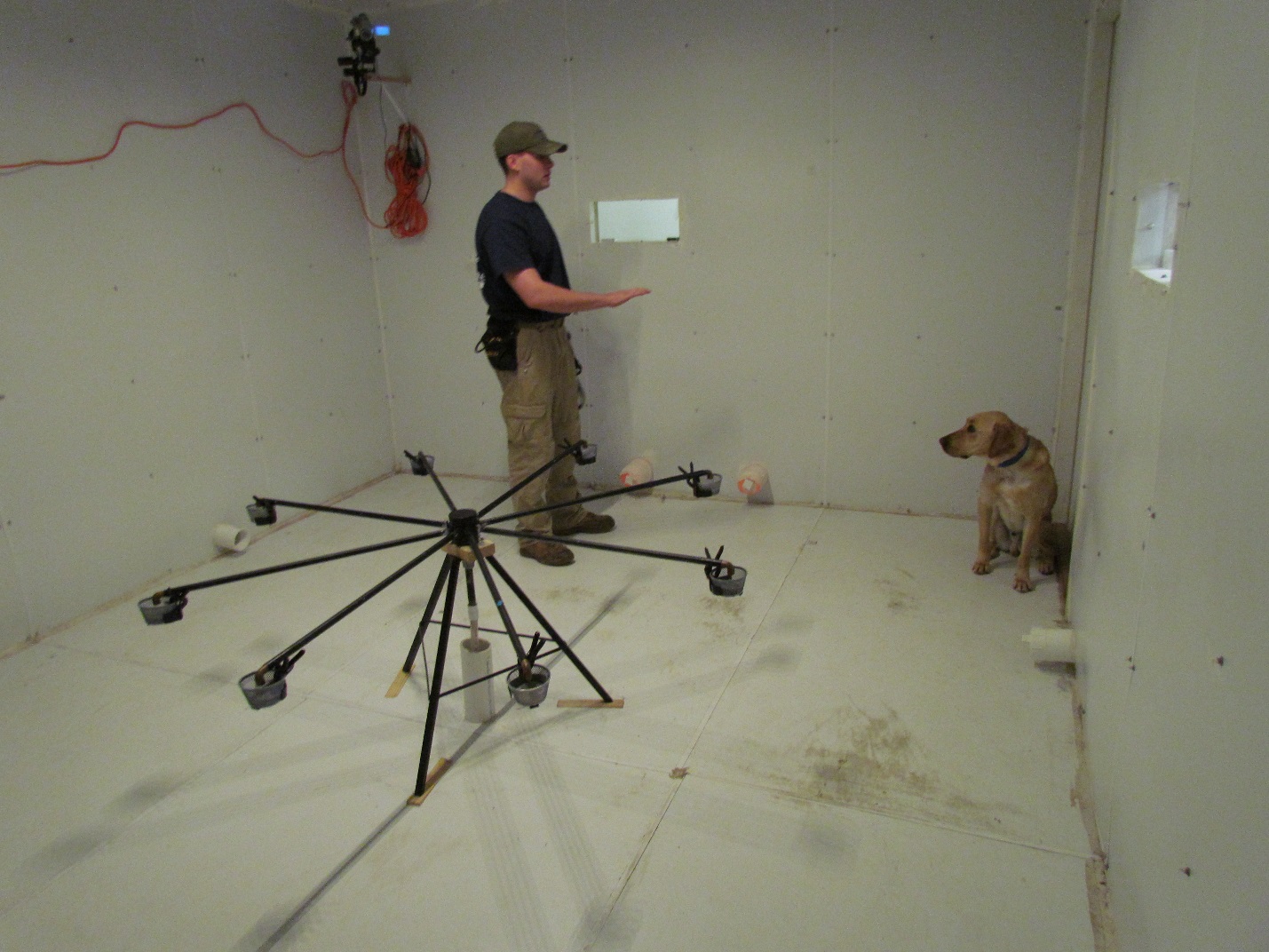


Supplementary Figure 1: Scent room with scent baskets representing arms 1-8 with handler signaling to dog before start of a search.
